# Formative Research to Design a Child-Friendly Latrine in Bangladesh

**DOI:** 10.3390/ijerph182111092

**Published:** 2021-10-21

**Authors:** Tarique Md. Nurul Huda, Tania Jahir, Sushobhan Sarker, Farzana Yeasmin, Abdullah Al Masud, Jesmin Sultana, Jyoti Bhushan Das, Fosiul Alam Nizame, Elli Leontsini, Abul Kasham Shoab, Laura H. Kwong, Mahbubur Rahman, Stephen P. Luby, Peter J. Winch

**Affiliations:** 1 Infectious Diseases Division, International Centre for Diarrhoeal Disease Research, Bangladesh (icddr,b), Mohakhali, Dhaka 1212, Bangladesh; tania.jahir@icddrb.org (T.J.); ssvn1991@gmail.com (S.S.); fyeasmin@icddrb.org (F.Y.); almasud@icddrb.org (A.A.M.); jesmin.sultana@icddrb.org (J.S.); jyoti.das@icddrb.org (J.B.D.); fosiul@icddrb.org (F.A.N.); akmshoab@icddrb.org (A.K.S.); mahbubr@icddrb.org (M.R.); 2 Department of International Health, Johns Hopkins Bloomberg School of Public Health, Baltimore, MD 21205, USA; eleontsi@jhu.edu (E.L.); pwinch@jhu.edu (P.J.W.); 3 Woods Institute for the Environment, Stanford University, Stanford, CA 94305, USA; kwong.laura@gmail.com; 4 Division of Infectious Diseases and Geographic Medicine, Stanford University, Stanford, CA 94305, USA; sluby@stanford.edu

**Keywords:** sanitation, latrine, Bangladesh, young children, child-friendly

## Abstract

In low- and middle-income countries, most latrines are not accessible to young children. We explored how to modify existing pit latrines to make them child friendly. We conducted four focus group discussions with mothers to explore barriers to child latrine use. We then enrolled 20 households with a child aged 3–7 years old to test six enabling technologies developed based on the identified barriers. Two to three weeks after installing the selected enabling technologies in each household, researchers conducted 19 in-depth interviews with caregivers to explore the technologies’ acceptance and feasibility. Common barriers included the discomfort of squatting on a large pan, fear of darkness, and fear of a slippery floor. Of the potential solutions, a ring to stabilize the child while squatting in the latrine was preferred by children and was affordable and available. A wooden board with a smaller hole than the usual pan reduced fears of falling and helped eliminate discomfort but was inconvenient to handle and clean. A transparent fiberglass roof tile was affordable, available, increased visibility, and kept the latrine floor dry. In conclusion, the fiberglass roof tile and stability ring were two affordable and locally available technologies that facilitated latrine use by children aged 3–7 years.

## 1. Introduction

Open defecation and the unhygienic disposal of feces facilitate the transmission of diarrhea-causing enteric pathogens by contaminating the environment [1,2,3,4,5]. Enteric pathogens can spread from the contaminated environment to children and adult family members and neighbors [6]. Compared to the hygienic disposal of feces in a latrine, unhygienic feces disposal in a drain, ditch, bush, or garbage heap increases the risk of diarrheal diseases [7,8]. Diarrhea is a leading cause of childhood mortality and morbidity, causing an estimated 477,000 deaths globally in 2016 [9,10]. Diarrheal diseases hindered the physical and cognitive development of young children and contributed to 72 million Disability-Adjusted Life-Years (DALYs) in 2015 [11,12].

In Bangladesh, 85% of households use improved latrines [13]. Despite the high availability of latrines and the low prevalence of open defecation by adults, open defecation is common among children in Bangladesh [14,15]. In rural Bangladesh, open defecation occurs inside the house or in courtyards [1,16,17]. Open defecation by children has also been identified in many other low- and middle-income countries, including India, Sri Lanka, Indonesia, Philippines, Burkina Faso, and Peru [7,18,19,20,21,22,23]. In Burkina Faso, more than 99% of mothers reported that children ages three and under do not use latrines [24]. Similar reports on children’s non-use of latrines were shared by mothers in Peru [25] and Bangladesh, where most children start using latrines around three to four years of age [17]. Children’s non-use of latrines adds to caregivers’ burdens as they must collect and dispose of children’s feces [7]. Many caregivers do not dispose of a child’s feces in a latrine in low-resource settings, even if they have access. In Cambodia, only 20% of the feces of children under five years old are disposed of in an improved latrine [26]. In Bangladesh, the safe disposal of children’s feces is low (44% in rural areas), and children’s feces are often disposed of in open areas or nearby bushes with leaves, straws, and paper [14,15,17]. In the nearby Indian state of Odisha, children’s feces are also often disposed of as household solid waste [20].

Since most latrines are designed for adults, they are rarely suitable for children. Parents of young children fear that children may injure themselves without proper supervision and assistance while defecating, such as by falling into the latrine hole or being bitten by rats or insects [23]. Parents also have concerns that poorly constructed latrines attract flies and emit odors that make them unpleasant [23]. One study in Cambodia found that a child’s age and the amount of the caretaker’s time required to supervise children while in the latrine were primary barriers [27]. A systematic review reported that latrine use depends on sanitation structure and design characteristics such as maintenance, accessibility, privacy, cleanliness, and whether or not the latrine was recently constructed [28].

Intervention efforts to increase latrine use typically focus on adults and neglect children’s needs [28]. There have been some efforts to promote child potties in order to improve the disposal of children’s feces in low-income countries [7,15,29]. However, there is a gap between the age when children stop using potties and when they start using latrines. This gap could be reduced if latrines were constructed with child-friendly design elements.

Enabling technologies can improve water, sanitation, and hygiene (WASH) behaviors [30,31,32]. The open defecation practices of children are hard to change [33]. Effective interventions must effectively address contextual, psychological, and technological factors that are barriers to safe disposal of children’s feces. Designs that incorporate improvements to make latrines child friendly could prove helpful in overcoming barriers to child latrine use, while simultaneously allowing caretakers to build trust in the technology and limit time spent with the child in the latrine [27].

“Universal design” or “design for all” is a concept in the literature regarding aging in high-income country contexts [34]. Universal design is defined as “an approach to creating environments and products that are usable by all people to the greatest extent possible” [35]. There are seven principles of universal design: (1) equitable use; (2) flexibility of use; (3) simple and intuitive use; (4) perceptible information; (5) tolerance for error; (6) low physical effort; and (7) size and space for approach and use [35]. Research regarding home modifications for elderly or disabled persons provides valuable insight into modifying latrines for children [36]. A limited number of studies have explored the utility of infrastructural supports in assisting older people’s use of latrines [37,38]. Elderly users appreciate the presence of hand supports, and good lighting as these make latrine use more comfortable [37,38]. Some of these concepts can be used to make latrines more accessible and usable for non-elderly people as well, particularly children, [34] and improve safe management of children’s feces in countries like Bangladesh.

This formative research study aimed to understand how to make existing latrines in rural Bangladesh user-friendly for children aged from three to seven years old. Children in this age group are mobile and are expected to transition from using the potty to using the latrine. But children in this age group are less likely to use latrines consistently than older children, even in households with access to a functional latrine [20,27]. Hence, we sought to develop a child-friendly latrine that would overcome children’s barriers to consistent latrine usage. Our study objectives were to (1) identify the reasons for children not using the latrine, (2) identify the barriers children face when using existing latrines, and (3) develop enabling technologies and behavioral recommendations to increase children’s latrine use.

## 2. Materials and Methods

### 2.1. Study Setting and Design

This study was conducted in rural areas of the Katiadi sub-district of the Kishoreganj district of Bangladesh. It is located a few hours from the capital of Bangladesh, Dhaka. We selected two villages (Char Betal and Adampur) out of convenience. Fishing and harvesting crops like rice, vegetables, lentils are the main sources of income for this region’s inhabitants. Houses are mostly built of bamboo, corrugated iron sheets, bricks, or combinations of these materials. Most households are extended families with an average of six members. An extended family consists of several generations and can include biological parents and their children, as well as in-laws, grandparents, aunts, uncles, and cousins. Our target population was children aged from three to seven years and their families because, in rural Bangladeshi settings, they are the primary caregivers.

The study was conducted in three phases: (1) focus groups, and (2) the first trial of improved practices (3) the second trial of improved practices (Table 1).

### 2.2. Guiding Theoretical Framework

We used the integrated behavioral model for water, sanitation, and hygiene interventions (IBM-WASH) to guide the qualitative data analyses and the interpretation of qualitative data, and to develop modified designs to make latrines child-friendly [32]. The IBM-WASH has been well established in a similar context [31]. The IBM-WASH identifies three dimensions: contextual, psychosocial, and technological factors [31]. The contextual level includes structural factors like climate, weather (e.g., rain), physical environment and space, the roles and responsibilities of family members, the structure of the household, and opportunities for and barriers to behavior formation [32]. The psychological level of the IBM-WASH model refers to behavioral and social factors such as culture, values, efficacy, injunctive and descriptive norms, knowledge, disgust, fear, and perception regarding a certain behavior or the adoption of a technology [32]. Injunctive norms refer to the perception of what should be, what is ideal, and what is approved or disapproved of in society. On the other hand, descriptive norms encompass existing practices in the community as they are perceived by others [39]. Technological factors influence the adoption of physical technologies in a community. Physical aspects, such the design, build quality, ease of use, price, and availability of technologies, play a definitive role in the adoption or failure of those technologies [32].

### 2.3. Focus Group Discussions

Our study team, trained in qualitative research, conducted four focus group discussions in two villages, with caregivers of 3–7 year old children, to understand the barriers children face when using existing latrines, the solutions that parents had developed, and the barriers to and opportunities for modifying existing latrines to make them child-friendly. Each focus group included three researchers with various roles: facilitation, note-taking, and gatekeeping. The role of the gatekeeper was to manage the crowds during the group discussions and to support the facilitator in managing the participants.

All focus groups were recorded using digital audio recorders. On average, each discussion lasted 45–50 min. Based on these findings, our team developed four enabling technologies and behavioral recommendations regarding latrine use by 3–7 year old children.

### 2.4. First Trial of Improved Practices

To understand the feasibility and acceptability of modifications that would make latrines child-friendly, we conducted two trials of improved practices (TIPs) [40,41]. Firstly, our field team listed 15 households from each of the two villages who had children between the ages of 3 and 7 years old and owned a functional latrine, but where the children did not use the latrine. From this list, we enrolled the first 12 households who agreed to participate in our study and received consent to modify their latrines. Next, our field team visited the identified households and assessed their latrines to determine suitable modifications based on the formative research findings.

During the FGDs we asked participants about the difficulties they and their children faced using latrines and sought their suggestions on how to overcome these difficulties. Considering the findings from the FGDs, the study team then brainstormed among themselves and developed four candidate toilet modifications that could address the barriers to children’s latrine use. The enabling technologies included: (1) a ring with a rope; (2) a wooden seat/board placed over the adult-sized latrine pan; (3) a laminated cartoon image with hygiene messages; (4) a transparent fiberglass roof panel. The details of how each prototype addressed the barriers identified are presented in the Results section. The team then worked with local carpenters to construct the technologies. The process of design and production took two months.

We tested four types of toilet modifications across all studied households. The modifications installed in a household were based on an assessment of the children’s ages and the condition of the latrine. There was a maximum of three modifications which were received by any one household. The households provided feedback on each of the modifications that they tested. Two weeks after making the modifications, the study team conducted an assessment to explore their feasibility and acceptability. Researchers conducted in-depth interviews with the caregivers/mothers and spot-checks at each of the 12 households to explore the advantages and disadvantages of the newly installed technologies, and to seek their suggestions to modify the technologies. Each 20–25 min interview was recorded using digital audio recorders to explore the participants’ opinions and experiences with the specific prototypes installed in their latrines. Interviews and spot-checks were conducted with pre-tested guidelines and checklists.

### 2.5. Second Trial of Improved Practices

Based on the feedback from the first trial of improved practices, we modified one of the enabling technologies (the wooden board with a handle) and added stairs as a new technology. In two new villages, the team enrolled 20 new households with children between the ages of 3 and 7 years old and at least one functional latrine for a second trial, with six modified and new prototypes. These households were selected based on the family members’ willingness to participate in the study, the need and scope of modification of their latrine, and their consent to conduct the latrine modifications. During household enrollment, the team planned for each prototype to be installed in at least one household latrine.

To assess the advantages and disadvantages of the installed prototypes in the second trial, we conducted 19 in-depth interviews. Six weeks after installation, the study team conducted semi-structured observations, followed by spot-checks, in 10 households. For structured observations, one researcher remained at a target household for 3–5 h. The observers used a semi-structured observation tool to collect data on the use of enabling child-friendly technology. If targeted events were not observed within the first 3 h, researchers were instructed to increase the observation duration to, but not surpassing, 5 h.

### 2.6. Data Analysis

Researchers summarized the recorded interviews from the audio recorder and translated the interview data into English for further analysis. During the data translation, the team carefully considered local terms and expressions. Researchers also translated and summarized their field notes, taken during interviews and focus groups, which contained details such as the respondents’ tone and attitude. The research team developed deductive codes based on the themes which were identified before data collection, using the interview guidelines and study objectives. Emerging (inductive) codes were also generated during data analysis. All the data were coded and categorized according to each tool used for the data collection: interview, semi-structured observation, and spot-check, and the team drew inferences from overarching findings.

The notes made during the semi-structured observation were then transferred into a Microsoft Excel worksheet, where data were compiled and sorted by both deductive and inductive codes. The research team then conducted a thematic analysis to consolidate the findings from the compiled data. These notes were also included in the thematic analysis. The findings from the focus groups were used to develop enabling technologies and accompanying behavioral recommendations. Interview and structured observation findings were used to inform the modifications of the proposed technologies.

### 2.7. Ethical Considerations

The study team obtained informed, written consent from participants in focus groups, interviews, and structured observations before data collection. The study protocol (PR-16037) was reviewed and approved by the institutional review board (IRB) of icddr,b, University of California Davis, and relied upon by the University of California Berkeley.

## 3. Results

### 3.1. Focus Group Discussions

A total of 32 caregivers participated in the FGDs; 19 were mothers, 4 were fathers, and 9 were grandmothers. None of the grandparents had any formal education. They could not read or write other than signing their names. Twenty-two parents completed 6–8 years of schooling, and one mother completed 12 years of schooling. Most of the participants reported that the mothers acted as the primary caregivers and managed their children’s bathing, feeding, and defecating. Participants also said that sometimes the grandparents and elder siblings helped with these tasks.

During the focus groups, 28 of the 32 participants responded that their children primarily used the latrine during the day and infrequently used the latrine at night; they never used the latrine during the rainy seasons. All participants (*n* = 32) agreed that their children needed their assistance while going to the latrine, especially at night. Mothers, grandmothers, and elder siblings primarily assisted the children in using the latrine and cleaning up afterward.

Almost all (*n* = 30) of the parents and grandparents reported that their concern was for safety when young children went to the latrine. Focus group participants mentioned that the children were hesitant to enter poorly lit latrines, even during the day (*n* = 30), and feared falling into the latrine pan (*n* = 12), falling due to slippery floors during the rainy season (*n* = 10), and entering the latrines alone (*n* = 7). Latrines were often installed in places that were poorly lit, even during the daytime. Sometimes, the latrines were elevated, making it difficult for children to enter the latrine without the caregivers’ assistance (Table 2). These concerns were especially relevant for children aged three to five years old.

One mother, age 28, who had completed secondary education, remarked:

*Children do not use the latrine because of fear. Very young children do not want to go to the latrine even in the daytime. Someone needs to accompany them, whether it is day or night.*

Another barrier the participants mentioned was the latrines’ distance from living quarters. Participants were asked about the distance and location of their latrines. Most (*n* = 30) reported that their latrines were built outside of the main household compound, with an average distance of 40 m from their living quarters. Latrines were often located near shrubbery, under a large tree, on the bank of a pond, towards the back, and in infrequently used places in the compound.

In these rural areas, mothers/caregivers did not want to go so far from their homes to assist their children in using the latrine, especially at night or during the rainy seasons. At these times, most children defecated either on the courtyard grounds or just outside the living quarters.

One grandmother, age 55, who had no formal education, reported:

*My grandson sleeps with me. In the rainy season, at nighttime, if he wants to defecate, I allow him to do it in the courtyard. It is risky for me to accompany him to the latrine crossing the muddy pathway in the dark of night.*

Most participants mentioned the increased chances of becoming sick with diarrhea or an upset stomach due to defecating in an open place, yet they continued to allow their children to practice open defecation because of the aforementioned barriers. Many participants mentioned the feeling of disgust and the bad odor as additional negative outcomes of open defecation.

One mother, aged 29, who had completed primary education, said:

*When I am busy in the kitchen, and my baby wants to defecate, I can’t accompany him to the latrine because that would require me to stop cooking. At that time, I allow him to defecate in the yard. I dispose of his feces when I finish cooking or before taking a bath. The feces emit a bad odor until I clean them.*

Participants also reported that none of their existing latrines were comfortable for children because they were made for adult-sized individuals. Squatting over an adult-sized latrine pan required children to spread their legs very wide, which was uncomfortable and sometimes painful. As a result, most children did not want to sit for a long time.

One 60-year-old grandmother stated:

*How can a little boy squat on an adult pan? It is painful for him to spread his leg to squat on a big pan.*

When asked about child-friendly strategies, the participants suggested improving the lighting inside the latrine. Most of them deemed it beneficial to build new latrines with child-friendly options rather than modifying the existing ones, because changing the pan of existing latrines had the risk of damaging the pit lining, making it unusable.

### 3.2. First Trial of Improved Practices

To address barriers reported in the focus groups and suggestions given by the participants, the research team developed four latrine modifications: (1) a ring with a rope to allow children to be more stable while they were squatting, reducing the fear of falling into the latrine pan; (2) a wooden seat/board placed over the adult-size latrine pan, to decrease the width between the foot placement areas and allow children not to spread their legs as wide; (3) a laminated cartoon image with hygiene messages, placed inside the latrine to attract the child’s attention to use the latrine; (4) a transparent fiberglass roof tile to increase the lighting inside the latrine (Table 3).

**Figure 1:**
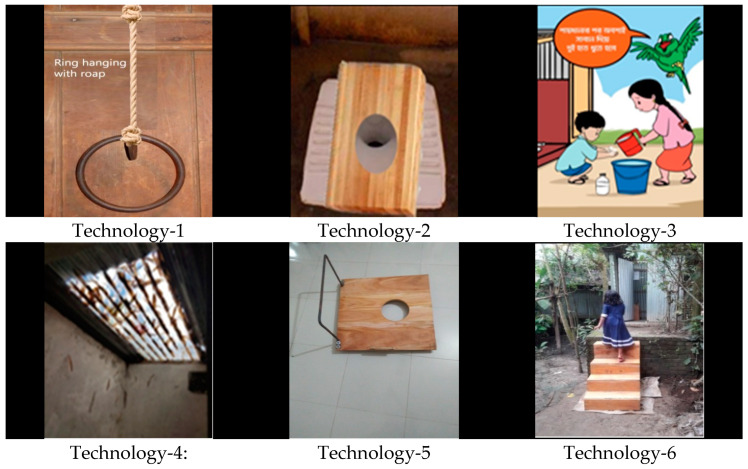
Enabling technologies from the two trials of improved practices. Technology-1: Iron ring hanging with rope inside the latrine to hold while squatting; Technology-2: Wooden seat placed on the pan to reduce the distance between children’s feet while squatting; Technology-3: Cartoon image with hygiene message to attract children inside the latrine; Technology-4: Transparent fiberglass roof tile to address children’s fear of the darkness; Technology-5: Wooden seat with the handle placed on the pan to make children sit comfortably; Technology-6: Stair to facilitate latrine access.

The study team installed four enabling technologies in 12 household latrines, while considering the participants’ preference and suitability of the latrine. Researchers installed a ring with a rope and cartoon image in all 12 households, provided a wooden board to five households, and replaced a corrugated iron roof with a transparent fiberglass roof in one household (Figure 1).

Two weeks after installation of the technology, the study team used interviews and spot-checks to assess the household’s experience using the technology. During the spot-checks, all 12 rings with a rope were placed correctly, but the study team could not confirm whether they were in use or not. All 12 images with the hygiene message were observed to be correctly placed and were clean and visible. All five of the wooden seats were found outside the latrine with no apparent sign of use. The transparent fiberglass roof was covered with tree leaves but undamaged.

During the in-depth interviews, 8 of 12 caregivers reported that the ring with the rope was preferred by the children, as they could independently hold onto it and use it to stabilize themselves as they squat, thereby reducing the fear of falling. Caregivers preferred the stabilization ring because it was affordable and could be made with materials available in local markets. Respondents were also asked about the adults’ reactions to having those in the latrine, and they reported none of the adult persons faced any problem due to having those technologies inside the latrine. Some disadvantages that they reported were that when the children played with the ring, it caused the roof, often made of bamboo or corrugated iron sheets, to move or shake, and sometimes the children felt pain in their hands and shoulders if they held onto the ring for a long time. One mother suggested that instead of a stabilizing ring, a moveable handle made of bamboo or wood could be installed that was in place while the child used the latrine and moved away when not in use.

Three of five mothers stated that the wooden board with a hole that exposed the latrine pan was effective in reducing the fear of falling and discomfort of sitting on the pan. Two mothers reported it was inconvenient to handle and clean, given its heavy weight. Two of the five mothers who had received a wooden seat suggested adding a handle for convenience. 

One mother, aged 25, said:

*It is very difficult to lift this heavy board. It is not easy to handle. Sometimes children are in a hurry, and we forget to place it. If there was a handle, we could easily lift it holding that handle.*

The transparent fiberglass roof tile improved visibility inside the latrine during the daytime and increased the evaporation of liquid on the latrine floor in the sunlight. Although the fiberglass roof was affordable and available, the one mother who had it installed in her latrine mentioned that if the latrine were located under a tree or bush, tree leaves would cover the fiberglass roof tile and block light from entering the latrine. She reported that cleaning the latrine roof was not easy, as it was difficult to climb up high enough. She needed to ask someone to help her clean the leaves off the roof.

All 12 mothers reported that the placing of an image of a cartoon character with hygiene messages inside the latrine captivated the children’s interest for the first few days; later, the image failed to attract the children’s attention. All 12 mothers reported that the picture was not available in the local market, and they did not think it could be made locally. Two mothers suggested adding a more interesting picture instead of the selected cartoon.

After a 5 h observations session, one mother shared:

*The picture was attractive to my son for 3–4 days, then he stood in front of the picture, observed it for a long time, and asked me questions about the content. After 3–4 days, he did not even look at it.*

Caregivers reported that, in some cases, children could not enter the latrine without adults’ assistance due to the height of the latrine floor. Latrine floors are often built higher than the yard to protect from flooding. Based on the feedback received from the first round of trials of improved practices, the wooden board was modified by adding a handle, and a new prototype was added, stairs (Table 4).

### 3.3. Second Trial of Improved Practices

In the second trial, the study team installed two to four different types of child-friendly prototypes in 20 households of two different villages: a ring with a rope (*n* = 16), a wooden board with a handle (*n* = 4), a wooden board without a handle (*n* = 4), the image of a cartoon character with hygiene messages, the same that was used in the first trial of improved practices (*n* = 20), a transparent fiberglass sheet on the roof (*n* = 15), and stairs placed at the entrance of the latrine to make the latrine accessible for children (*n* = 1).

Three weeks after the installations, our team conducted interviews with 19 mothers. One mother was absent during data collection and was not able to be included. Three-quarters (*n* = 12) of the mothers, assigned the ring with a rope, liked the technology because their children could hold the ring during defecation and control their physical balance comfortably with minimal assistance from mothers.

One mother reported:

*Earlier, I needed to accompany my baby, leaving urgent household chores. He used to hold my hand while sitting on the pan to sit comfortably. After installing this ring in our latrine, now he can hold it easily and does not ask me to assist him.*

Eleven out of sixteen mothers reported that this prototype was feasible and acceptable, noting its availability and low cost. They mentioned that it could be made at home with existing materials. The mothers suggested no further modifications for this prototype and added that the elders benefitted from this enabling technology as well.

The study team provided a wooden board with a handle to four different households. Mothers said that they did not like the wooden board as it was difficult to clean and too heavy to handle (Table 4, Appendix A Table A1).

Half (*n* = 10) of the respondents who received a cartoon with hygiene messages said that they liked the image and that it attracted their children to enter the latrine. Some children were very curious to see the image; they observed it closely both during and after using the latrine. They asked their mothers about the meaning of the pictures and the hygiene messages. Some (*n* = 4) mothers said that their children went to the latrine only because of the picture and that their children started washing their hands after using the latrine for a few days, after their mother taught them about handwashing based on the picture. They suggested adding additional hygiene information to the image so children could learn more. A few mothers said that their children were interested in the picture for the first few days, but this interest quickly waned. Most (*n* = 12) mothers found the image acceptable and feasible to maintain, as it was easily cleaned every three to five days (Table 4).

All 15 respondents who were provided with the transparent roof panel reported that they were satisfied with the transparent roof. They reported that the transparent roof allowed light inside the latrine, reduced their electricity bill as they did not need to turn the lights on during the daytime, kept the latrine floor dry, reduced the flies present in the latrine, and addressed concerns about falling on a slippery floor. They also reported that the presence of sunlight reduced any bad odor and even decreased adults’ feelings of disgust at using the latrine (Table 4).

One respondent, who was 35 years old and had primary education, stated:

*My mother-in-law was scared of falling due to a slippery floor. Now she can use the latrine without fear, as it remains dry due to the sunlight passing through the transparent roof. I would recommend this not only for children but also for elderly persons in the family.*

One difficulty faced by the mothers was the issue of cleaning the roof when it became covered with tree leaves. All participants (*n* = 15) said it was not a major problem, and most (*n* = 11) reported that this problem could be solved easily by removing the leaves from the roof. The women felt they could not remove the leaves themselves, but they could ask a male household member to clear off the roof. There were no additional suggested changes for this prototype, which suggested that it was sufficiently easy to use and maintain.

One household tested the stairs; they were appreciated by the family members and the child who used it, and they did not face any disadvantages when using the stair. The mother reported that in addition to the children, the stairs were helpful for the elderly person of the family, as it made climbing the latrine easier when compared to having to climb the high latrine surface. The mother suggested making the stairs with brick and cement instead of wood to increase their durability.

The mother who received the stairs, aged 32 with no formal education, said:

*As it is (stair) made of wood, it will be damaged soon because it is placed under the open sky. Bright sunlight, heavy rainfall, fog can cause harm to it. If you could provide stairs made of concrete/brick and cement, that will last for a long time.*

The mothers stated that none of the child-friendly hardware inside of the latrine hindered comfortable latrine use for other family members. The mothers noted that it took approximately two weeks for the children to become acquainted with the newly installed technologies. They mentioned that the technologies helped growing children develop the habit of latrine use. Among other technologies, the wooden board, stairs, and the cartoon image with messages were not readily available in the market but could be made or customized locally. Community residents considered the price of these interventions and expressed a willingness to purchase and install them.

Respondents were then asked about their preferences among all enabling technologies. When considering the advantages and disadvantages of each prototype, they ranked “fiberglass roof tile” as highly preferred and “wooden seat with/without a handle” as the least preferred option.

The team observed four of ten children using the latrine. All four children were found using the ring with the rope comfortably. One of four children used the wooden seat and seemed uncomfortable using it.

Except for the wooden board, with or without the handle, each spot-check confirmed that the provided interventions were in the correct place, appeared to be regularly cleaned, and regularly maintained by caregivers. The wooden board was not found close to the latrine.

## 4. Discussion

This formative pilot study demonstrated that modest modifications to a latrine, including adding a ring with a rope for a child to hold onto, a cartoon image with hygiene messages, and substituting transparent fiberglass for a corrugated iron sheet roof, were acceptable to latrine users and addressed major barriers to children using adult latrines in a village in Bangladesh.

Through this study, researchers identified acceptable and feasible child-friendly interventions using the IBM-WASH framework for household latrines in rural Bangladesh. The factors associated with latrine use by children are discussed here according to the three dimensions identified in the IBM-WASH framework (Appendix A Table A2) [32].

Contextual factors: In rural Bangladesh, mostly the mothers of the children were found acting as the primary caregivers who oversaw their children’s defecation routines and were sometimes aided by grandparents and older siblings [33]. That is why the primary target of our intervention was mothers and other caregivers, including grandmothers and elder siblings. Various individual-level factors, such as age, influence child latrine use. The children’s ages were noted as an influential factor in latrine use, as younger children aged between three to five years old could not go to the latrine on their own. At this young age, children are usually assisted by other family members. For this reason, we considered recommending building latrines close to the house. However, it was not feasible within this study to build new latrines close to home. Future studies should consider the feasibility and acceptability of building pit latrines close to the main house in a rural setting. It is possible that building latrines closer to home may attract more flies to the food preparation area. Therefore, future studies should consider ways to build latrines closer to homes, but away from food preparation areas. In developing the child-friendly modifications, we considered the children’s ages and ensured that the hardware added to the existing latrine was suitable for use by young children. In addition, the proposed interventions included recommendations to assist young children in using the latrine until they are fully trained in use of the latrine, with the new child-friendly technologies, by themselves. 

In rural Bangladesh, most of the responsibility for childcare falls to the mother or other women in the household. Women and girls have a larger role relative to men in water, sanitation, and hygiene activities, including in agriculture and domestic labor [42,43]. However, these interventions have the potential to encourage children to use the toilet independently, which would mean less work for female caregivers, especially tasks like cleaning up feces from the household premises. This formative research focused on enabling technologies and did not adequately explore how the installation of these technologies may impact women’s workload, or how to change these gender norms. Future studies should consider how the responsibility of toilet training can be shared between parents and other household members to reduce the burden on women, in addition to structural modifications such building toilets near the home.

Environmental factors, such as the time of day and seasonality, also played a significant role in obstructing child latrine use. Since the latrines were located far from the house, children did not want to visit them at night. During the rainy season, slippery mud made the journey to the latrine difficult even during the daytime; as a result, it was not even possible for their caregivers to assist them to the latrine. The proposed interventions, the ring attachment, the transparent fiberglass roof, and the wooden board, resolved squatting problems, reduced the fear of falling, of darkness, and/or the slippery floor. These interventions improved the physical environment and enabled children to go to the adult latrines safely.

Psychological factors: Young children defecating in the courtyard, especially at night and during the rainy seasons, was the descriptive norm in our study, although mothers and caregivers did believe that latrine use was more hygienic. However, existing latrines were not child-friendly and often went unused.

Disgust was a strong factor at the psychological level. Caregivers had to handle the wooden board directly, so they felt disgusted to move the board or to clean it. In our trials, the wooden board was not well-received because it was not easy to carry, lacked a handle, and often went unused by families.

The ring and the transparent roof tile, which were added to existing latrines, motivated and enabled caregivers to improve child defecation practices. The promotion of behavioral recommendations associated with the enabling technologies improved caregiver knowledge about safe defecation and the hygienic practices of children, and translated injunctive norms into daily action, a core goal of our study.

In our study, the cartoon image messages depicting hygienic latrine rituals and handwashing behaviors piqued interest among users initially. The cartoon image with the hygiene messages had the potential to promote handwashing habit formation [44,45]. However, in this study, we did not assess the impact of this intervention on handwashing habit formation. Household members did not believe this was a feasible intervention, given that the pictures were not readily available to purchase locally.

Technological factors: The stabilization ring with the rope was widely accepted because of its availability, simple construction, and inexpensive materials. The children found the ring and rope easy to use as well. The fiberglass roof was locally available, easy to install and maintain, and inexpensive to purchase. The transparent roof allowed sunlight into the latrine, which helped keep the latrine floor dry and well-lit during the daytime. Thus, the transparent roof reduced the fear of darkness and helped prevent slipping on the floor, benefiting both children and adult users. It is also possible that the improved lighting also improved adult aim at the latrine, thereby improving cleanliness. The wooden board without a handle was not well accepted in the first trial of improved practices, so we modified it by adding a handle. Even with improvements in the second phase, it was not accepted by the community.

We determined that the transparent fiberglass roof panel, a ring with a rope, and stairs were convenient, affordable, safe, and acceptable facilitators for use of the latrine for defecation by young children. These interventions saved the caregivers’ time and reduced the burden of labor they face when disposing of children’s feces, while motivating and enabling adults to educate their children on proper, hygienic latrine use regularly.

The barriers to latrine use identified in this study were similar to other studies conducted in other low-resource contexts [27,33,46]. Common barriers included the distance to the latrine, the discomfort of squatting on a large pan, fear of darkness, and fear of a slippery floor, among others. In Cambodia, a study explored the current practice of infant and young children’s feces management, and found that children aged five and under needed assistance from others to use the latrine because they were afraid of falling and could not sit comfortably on the adult-sized pans [27]. A study conducted in urban Uganda found that the lack of well-marked paths and adequate lighting in the latrine cubicle were barriers to latrine use at night [46].

This formative research started with a trial of improved practices to introduce sample technologies to the population. For this reason, we do not have an accurate estimation of whether households could be convinced to purchase these technologies outside of a study under routine programmatic conditions in the future. Nonetheless, our study findings provide important insights regarding the acceptance, use, affordability, and availability of the technologies promoted. Additionally, this study engaged a small sample of households, so we do not know how well-received these interventions would be at the level of an entire sub-district or district. However, the study was conducted in areas that are typical of rural Bangladesh in terms of economic productivity and access to water, sanitation, and hygiene services. We did not examine the sustained adoption of new behaviors before, immediately after, or many months after the installation of the technologies. The seasonal barriers, such as the use of latrines in rainy seasons, also remained unresolved, as none of the technologies evaluated had fully addressed the barriers to use during the rainy season. The study team attempted to select study households that represent average rural Bangladeshi households in regard to household structure, household wealth status, and cultural views; however, there may be subgroups that would not find the technologies evaluated to be acceptable. Even with the small sample size, this study was able to show the feasibility and the acceptability of modifying latrines to make them child friendly. In this study, feasibility and acceptability were measured based on the mothers’ reports. Given that this study provided the enabling technologies, it is possible that the mothers’ responses were influenced by courtesy bias. However, some of the findings were confirmed by observation. We received both negative and positive feedback from the respondents. Distance to the latrine was an important barrier mentioned by the mothers; since in this study we only focused on changing the existing latrine, we did not have the scope to address this barrier. Addressing this issue of the distances to latrines will require an in-depth understanding of the reason for building latrines away from the living quarters, and if building latrines close to the main house is feasible in the rural context.

Our study used the IBM-WASH model to identify different contextual, psychological, and technological factors that may influence latrine use among children. We considered these factors at the habitual, behavioral, individual, and interpersonal/household levels of IBM-WASH (Table A2). Contextual factors such as the gender of the primary caregiver, their role and position in the household, the contribution of other family members in child caregiving, and the children’s ages were considered when designing the interventions. Environmental factors such as the time of day, seasonality, and the physical environment/condition of the latrines have a significant role in determining latrine use by children, so these factors also need to be considered. At the psychological level, disgust was identified as a strong factor in using and maintaining toilet modifications. At the technological level, factors such as the availability and cost of the materials used for latrine modifications, visible benefits of the modifications, were identified as important factors in the acceptability of these toilet modifications. Future studies should consider these factors identified in this formative study in the design of interventions and assess whether these factors were addressed in the design of child-friendly modifications. Future studies should consider longitudinal study designs to understand how modifications to latrines to make them child-friendly improves the health and quality of the lives of children and their parents in resource-constrained settings.

## 5. Conclusions

Enabling technologies combined with appropriate behavioral recommendations are the cornerstones of effective sanitation programs. These study findings demonstrate the feasibility of addressing barriers to the use of existing latrines by children and of making them more child friendly. Innovative technologies create environments to enable behavior change but are limited by access, availability, cost, and physical characteristics, such as design, weight, and size. Further research on the effectiveness of child-friendly latrines in reducing open defecation would help our efforts to improve sanitation access for all. Specifically, future studies should explore if adding stabilizing ropes, transparent roofs, and stairs increases the use of latrine by young children. These modifications to latrines can be added when a new latrine is installed or when suitable, for modifying existing latrines. In addition to installing technologies, we recommend that mothers and caregivers receive regular training on how to train young children to use latrines and to sustain this use over time. Interventions that combine the installation of child-friendly latrines with latrine training may reduce the gap in time when children stop using potties and start using the latrine. Further research may also examine how installing new, child-friendly latrines closer to the main house affects latrine use by children and the safe disposal of children’s feces.

## Figures and Tables

**Table 1:** Data collection methods.

Focus Group Discussions		
Methods	Respondents	Objectives
Focus group discussions Duration: 40–50 min	2 villages were selected by convenience 4 FGDs (8 participants per group)	Understand barriers children face when using latrines. Identify community or parent-led solutions. Make or modify latrines for child-users.
**The First Round of Trial of Improved Practices**		
**Methods**	**Respondents**	**Objectives**
In-depth interviews Spot-checks	12 households from two villages who consented to latrine modifications. Households’ children aged 3–7 years with a functional latrine that was not being used by children.	Explore the feasibility and acceptability of installed technologies Seek community input on prototypes and modifications. Conduct spot-checks to assess adherence to guidelines
**The Second Round of Trial of Improved Practices**		
**Methods**	**Respondents**	**Objectives**
Needs assessment	40 new households with latrines from identified villages	Understand what types of modifications or installations would benefit families with latrines
In-depth interviews	19 interviews were conducted from two villages. Households with children aged 3–7 years with a functional latrine that was not being used by children.	Assess the advantages and disadvantages of the installed prototypes in the second trial
Observations	10 structured observations (5 from each village).	Noting useful events, explanations by the households, and individual observations.

**Table 2:** Key findings from the focus groups.

Problem Identified	Proposed Solutions
Children scared of the darkness	Ensure the latrine is well-lit
Children scared of going alone	Parents can accompany children for the first few days until the child becomes habituated
Children fear falling into the pan	Add a handle or railing inside the latrine that the child can hold during defecation
Children can slip on wet floors or latrine pans during the rainy season	Ensure the latrine has a roof
Inside of the latrine cubicle is too unattractive for children, so they do not like using it	Place a cartoon on the wall inside the latrine, so the mother can point to it and tell her child a story while the child defecates
Squatting on a large pan that is designed for adult use is uncomfortable for children	Add a wooden board to effectively reduce the size of the latrine pan

**Table 3:** Accompanying behavioral recommendations for each of the latrine modifications.

Latrine Modifications	Behavioral Recommendations
Ring with Rope	Assist your children in holding the ring during sitting on the pan, and s/he will not fall down If the rope is ragged, then tie the ring with a new rope Assist your child while using the latrine until they are habituated to using this
2. Wooden seat/board	Place the board on the pan for children to use while they squat Remove the board from the pan after s/he completes defecation Wash the board when it is soiled/needed Assist your child while using the latrine
3. Cartoon image with hygiene messages	Children will see the image while using the latrine, s/he will enjoy it Clean it when needed If it is misplaced, hang it again in a suitable place Assist your child while using the latrine
4. Transparent fiberglass roof tile	Clean the fiberglass roof tile if it is covered with leaves so that light can enter the latrine Assist your child while using the latrine
5. Wooden seat/board with handle	Place the board on the pan, and children will sit on it and will hold the handle during defecation Remove it from the pan after s/he completes defecation Wash it when needed Assist your child while using the latrine
6. Stairs	Assist children while climbing the stairs Assist your child while using the latrine

**Table 4:** Responses of users to six enabling technologies for child-friendly latrines.

Prototypes	Advantages	Disadvantages
Ring with the rope	No fear of falling into the latrine pan	Children feel pain in hands after holding for some time
2. Wooden seat	Children can sit comfortably Reduces fear of falling into the latrine pan	Heavy, so caregivers find it difficult to move in and out of the latrine whenever a child might need it Needs an extra place to store it Difficult to clean Difficult to hold for moving
3. Picture of a cartoon character with hygiene-related behavioral recommendations	Children like the image Does not require frequent maintenance Easy to clean	The poster does not currently exist in the local market Not interesting to children after a few days
4. Transparent fiberglass roof tile	Increases light inside the latrine Reduces children’s fear of the darkness	Need to clean it frequently to allow the lights in, especially on stormy or windy days Female members cannot clean the roof
5. Wooden seat with handle	Children can sit comfortably Reduces fear of falling into the latrine pan Holding the handle provides additional stability while squatting	Heavy, hard to lift Needs an extra place to store it Difficult to clean
6. Stairs	Children can access the latrine alone	Considering durability, stairs can be made of brick and cement instead of wood

## Data Availability

The data that support the findings of this study are not publicly available because we did not ask participants to consent to raw data sharing outside of the research team. Public sharing of the data could compromise anonymity and research participant consent.

## Appendix A

**Table A1:** Key findings from two rounds of Trial of Improved Practices.

Technology	Advantages	Disadvantages	Affordability	Recommendations
1. Ring with the rope	Children can hold it and sit alone; it has reduced their fear of falling down	Sometimes children complained that by holding this ring they feel pain in their hands Children use it for hanging and playing	It is readily available and cheap, but most people might not be willing to pay to be purchased from outside	Replace the ring with any movable handle that will move according to the baby’s need
2. Wooden seat	The wooden seat is flat, and the hole is small, that’s why children can sit on it comfortably	First 2/3 days they used it willingly but after that mother threatened him /her if s/he does not sit here then the police will arrest him/ her parents Needs to be placed by an adult before children must use it For its heavy weight, mothers faced difficulty moving it	It is not readily available but can be made from a carpenter’s shop and is affordable, but most people might not be willing to pay	The wooden seat should have a handle to move it as needed Instead of wood, plastic can be used to make the seat, then it will be lightweight. It will reduce the cost also
3. Wooden seat with handle	The wooden seat is flat, and the hole is small, that’s why children can sit on it comfortably. Easy to use holding the handle	Heavy and needs an extra place to store it Not easy to wash due to weight and feelings of disgust	It is not available but can be made from a carpenter’s shop and is affordable, but most people might not be willing to pay	Instead of wood, plastic can be used to make the seat, then it will be lightweight. It will reduce the cost also
4. Picture of a cartoon character with hygiene related behavioral recommendations	Children also want to wash their hands after coming from the latrine One baby went to the latrine only to see the picture Most of the mothers said that the image was very helpful, and the children were interested to see this picture during defecation. No burden of maintaining like cleaning, washing	Out of curiosity, children look at it for the first 2/3 days, then they do not look at it Some mothers reported that children felt interested to see this picture for a few days. After a certain period, they did not feel interested	Almost all the respondents said that they had not seen such a picture ever and they do not know where they can get it They do not have an idea about the cost	More attractive pictures can be added Some of them suggested that, if the picture can be compiled with some different types of informative messages, then children can learn more things and can practice them
5. Transparent fiberglass roof tile	Reduces the darkness inside the latrine, and reduces the fear of children from darkness	Tree leaves fall onto it and cover the tile. Need to clean it frequently. Women cannot climb up on the roof easily. A male person can do it. As a result, if there is any storm the tile covered with leaves quickly	It is available and affordable and cheap, but most people might not be willing to pay	No suggestion A few of them asked, who have a concrete roof on their latrine and facing darkness, what can be done?
6. Stair	They are very happy to get this prototype because now their children are going to the latrine alone and feel comfortable	There was no disadvantage of this prototype because it is a matter of comfortably using the latrine	It will be very costly as it is made of wood Not affordable for all	The mother said that if the stair is made of concrete brick then it would be more durable, because the wooden stairs would be damaged quickly due to heavy rain

**Table A2:** The full IBM-WASH framework applied to the use of child-friendly prototypes.

Levels	Contextual Factors	Psychosocial Factors	Technology Factors
**Societal/Structural**	Rainy and winter seasons and their effect on child defecation habits	Leadership/advocacy for use of child-friendly technologies	Manufacturing capacity for child-friendly technologies
**Community**	Access to latrines	Shared values, collective efficacy for community-wide use of child-friendly technologies	Availability and distribution of child-friendly technologies in the community
**Interpersonal/Household**	Household members and division of labor related to childcare and disposal of child’s faces; condition of the latrine	Injunctive norms, descriptive norms for use of child-friendly technologies; responsibility for maintaining friendly technologies	Sharing of access to the product, modeling/demonstration of the use of the product
**Individual**	Wealth, education, and employment of caretaker of the child; age and developmental stage of child and their effect on the use of child-friendly technologies	Self-efficacy for the training of child and correct use of child-friendly technologies; knowledge of diarrheal diseases; disgust and perceived threat related to child feces in the household or courtyard	Strengths and weaknesses of child-friendly technologies for end-users; adaptation of design to respond to consumer preferences
**Habitual**	Favorable environment for habit formation on child-friendly technology use	Existing habits for disposal of child’s faces; outcome expectations: What is the expected outcome of using child-friendly latrines by children	Ease/Effectiveness of routine use of child-friendly technologies need for training; visibility of the technologies as a cue to action for the use
